# Temperature and composition effects on fresh and hardened properties of slag-fly ash-silica fume alkali-activated grouting materials

**DOI:** 10.1038/s41598-025-33462-0

**Published:** 2025-12-30

**Authors:** Yu-chen Qian, Li Zhang, Wei-guo Qiao, Yan-zhi Li, Yue Wu, Yun-rui Zhao

**Affiliations:** 1 Shandong Provincial Key Laboratory of Civil Engineering Disaster Prevention and Mitigation, Shandong University of Science and Technology, Qingdao, 266590 China; 2 College of Civil Engineering and Architecture, Shandong University of Science and Technology, Qingdao, 266590 China; 3 Taishan Vocational and Technical College, Taian, 271000 China

**Keywords:** Alkali-activated grouting materials, Slag-fly ash-silica fume, Curing temperature, Fresh and hardened properties, Microstructural evolution, Chemistry, Engineering, Materials science

## Abstract

Growing concerns over carbon emissions from traditional cement have intensified interest in alkali-activated materials (AAM) as sustainable alternatives. In grouting applications, key performance parameters such as strength, fluidity, viscosity, setting time, and bleeding rate are strongly influenced by both material composition and curing temperature. As underground engineering projects extend deeper, environmental temperatures gradually increase from ambient to 60 °C with depth, yet limited room-temperature studies on alkali-activated grouting materials (AAGM) cannot fully meet engineering requirements across temperature conditions. This study systematically investigated the effects of temperature (20 °C, 40 °C, 60 °C) and composition (including precursor ratios, activator modulus, and liquid-to-solid ratio) on the behavior of ternary slag-fly ash-silica fume AAGM. A comprehensive suite of characterization techniques, including XRD, FTIR, TG–DTG, SEM–EDS, ICC, NMR, MIP, and pH measurement, was employed to elucidate reaction behavior and microstructural evolution. Results indicated that elevated curing temperatures significantly accelerated early-age strength development and reduced setting time but decreased fluidity and increased viscosity. Microstructural analysis revealed enhanced precursor dissolution and the formation of more polymerized amorphous aluminosilicate networks at higher temperatures, accompanied by increased porosity and microcracking due to drying shrinkage. NMR results suggested a temperature-induced shift from low-coordination (Q^1^, Q^2^) to higher-coordination (Q^3^, Q^4^) silicate species, indicating increased binder polymerization. The study proposed suitable parameter ranges and provided insights into temperature and composition mechanisms, facilitating AAGM formulation optimization under specific temperature conditions.

## Introduction

Traditional cement-based grouting materials are extensively utilized in underground engineering because of their accessibility, economic viability, and well-demonstrated capability in enhancing mechanical properties and impermeability of fractured rock masses^1,2^. However, the substantial carbon footprint together with the high energy use linked to ordinary Portland cement (OPC) production have become increasingly incompatible with global carbon reduction targets. The production of 1 ton of OPC generates approximately 0.9 tons of CO_2_, accounting for about 8% of man-made CO_2_ emissions worldwide^3^. This environmental challenge, coupled with the growing demand for sustainable infrastructure solutions, has motivated intensive research into low-carbon alternatives.

Alkali-activated materials (AAM) have emerged as promising sustainable alternatives to OPC, delivering comparable or superior mechanical performance while reducing CO_2_ emissions by 60–90%^4^. These binders are synthesized by activating aluminosilicate precursors within a highly alkaline environment. During activation, the high concentration of OH^−^ ions creates a strong alkaline environment that attacks the vitreous structure of the precursors. This process redistributes electron density around silicon atoms, increasing the susceptibility of Si–O–Si and Al–O–Si bonds to breakage, thereby promoting the dissolution of the glassy phase and the release of monomers^5^. These monomers subsequently undergo polymerization and condensation to form a hardened calcium (alumino) silicate hydrate (C–(A)–S–H) or sodium aluminosilicate hydrate (N–A–S–H) gel network^6,7^.

To optimize the properties of this gel network for specific engineering applications, the selection of precursors is critical. While single-precursor systems are common, the formulation of ternary systems enables performance optimization by exploiting the synergistic effects of different precursors, addressing the limitations inherent to individual materials. For instance, ground granulated blast furnace slag (GGBFS), enriched with CaO, facilitates rapid setting and high early strength^8^. However, pure slag systems often exhibit poor flow retention. Fly ash (FA), characterized by its spherical geometry and lower reactivity, improves the workability of the fresh grout and contributes to later-age strength and thermal stability^9^. Silica fume (SF), with its ultrafine particle size and high pozzolanic activity, acts as a micro-filler to refine the pore structure and enhance the density of the interfacial transition zone^10^. Although ternary GGBFS–FA–SF systems have demonstrated promise in structural concrete, their optimal composition specifically for grouting applications remains adequately investigated.

In grouting applications, materials must satisfy strict requirements beyond high compressive strength, including controllable setting time, high fluidity, low viscosity, and minimal bleeding to ensure effective penetration into fractures^11,12^. These properties are significantly influenced by precursor proportions, liquid-to-solid ratio (l/s), activator modulus (Ms), and alkali content^13–15^. Furthermore, curing temperature constitutes a critical factor in deep underground engineering. Under the influence of geothermal gradients, the in-situ temperature in underground projects such as deep railway tunnels and mining roadways can range from ambient temperature to 40–60 °C^16,17^. Elevated temperatures generally enhance precursor dissolution, accelerate reaction kinetics^18^. However, existing literature primarily focuses on AAMs cured at ambient temperature or under high-temperature steam curing for precast elements. There is a specific research gap regarding the behavior of alkali-activated grouting materials (AAGM) under the variable moderate temperature conditions (20–60 °C) typical of deep underground environments. Specifically, the trade-off between temperature-induced strength gain and the potential risks of rapid fluidity loss and microstructural coarsening requires systematic investigation.

To address this research need, the present study examines how curing temperature (20 °C, 40 °C, 60 °C) alongside key compositional parameters, GGBFS proportion, FA/SF ratio, liquid-to-solid ratio, activator modulus, and Na₂O content, affect the properties of ternary slag–fly ash–silica fume AAGM. Both fresh properties such as setting time, fluidity, viscosity, and bleeding, and hardened properties like compressive strength were evaluated alongside comprehensive multi-scale microstructural analyses using X-ray diffraction (XRD), Fourier-transform infrared spectroscopy (FTIR), thermogravimetric and derivative thermogravimetric (TG–DTG) analysis, scanning electron microscopy-energy dispersive spectroscopy (SEM–EDS), mercury intrusion porosimetry (MIP), and nuclear magnetic resonance spectroscopy (NMR). Additionally, isothermal conduction calorimetry (ICC) and pH measurements were conducted to monitor reaction kinetics and alkalinity development. The findings are expected to clarify the relationship between curing temperature, material composition, and AAGM performance, and provide valuable guidance for material formulation design in underground environments with varying temperature conditions.

## Materials and experimentation

### Materials and specimen preparation

**Fig. 1:**
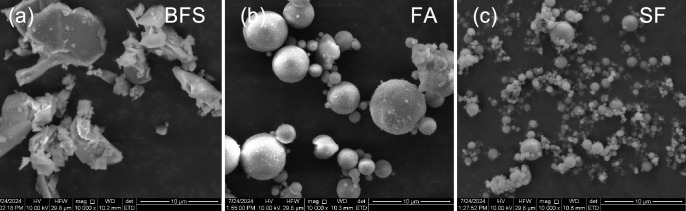
Electron micrographs of (**a**) GGBFS; (**b**) FA and (**c**) SF.

GGBFS (S105-grade), FA, and SF served as binder materials for this investigation. SF and FA were supplied by Henan Borun Foundry Materials Co., Ltd., while GGBFS was purchased from Gongyi Longze Water Purification Materials Co., Ltd. Microscopic examination revealed that GGBFS exhibited irregular morphology, whereas FA and SF appeared as spherical glass microbeads, as shown in Fig. 1. This morphological difference is critical for grouting performance; the “ball-bearing effect” of the spherical FA and SF particles effectively reduces inter-particle friction, thereby improving the fluidity of the fresh grout, which is essential for penetrating narrow fractures^19^. Conversely, the angular GGBFS particles provide a higher specific surface area for reaction but may increase viscosity.

**Fig. 2:**
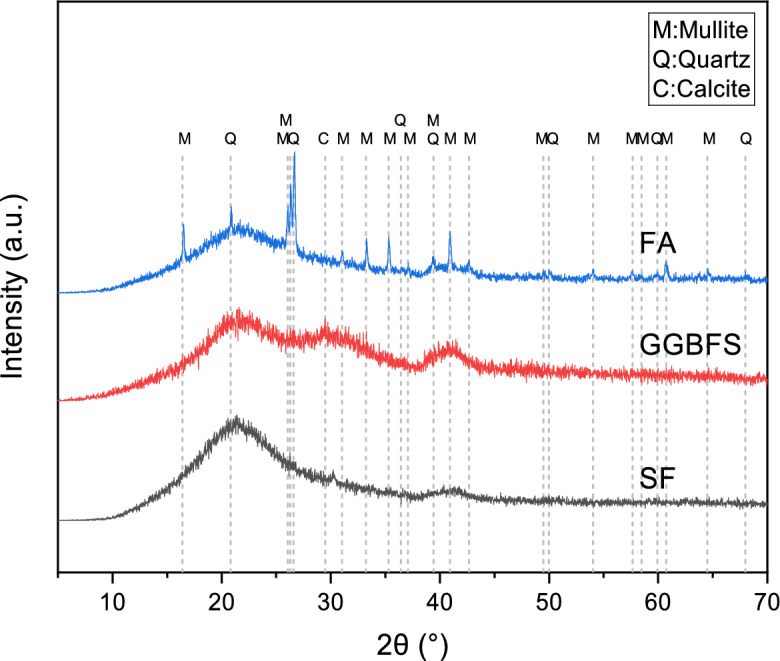
XRD spectra of raw materials.

Table 1 along with Fig. 2 present elemental compositions of these binder materials. Hydraulic modulus (HM) of GGBFS, determined as (CaO + MgO + Al_2_O_3_)/SiO_2_, was 1.65 (> 1.6), indicating high reactivity potential^11^. XRD analysis (Fig. 2) showed that FA contained crystalline peaks corresponding to mullite and quartz, while GGBFS and SF exhibited fewer crystalline peaks. The broad diffraction hump of GGBFS spanned from 20° to 40°, reflecting a high content of amorphous calcium aluminosilicate glass, which is the primary source of reactivity for early strength development. SF showed a broad peak around 20°–25°, indicating its highly reactive amorphous silica nature, which facilitates the refinement of the pore structure through the pozzolanic reaction.

**Table 1:** Chemicalcomposition in GGBFS, FA and SF.

Material	SiO_2_	CaO	Al_2_O_3_	SO_3_	Fe_2_O_3_	MgO	Na_2_O
GGBFS (wt%)	34.50	35.30	16.70	1.24	1.50	5.01	0.82
FA (wt%)	52.32	6.21	28.07	1.45	0.99	1.90	2.11
SF (wt%)	98.1	0.24	0.16	–	–	0.75	0.22

**Fig. 3:**
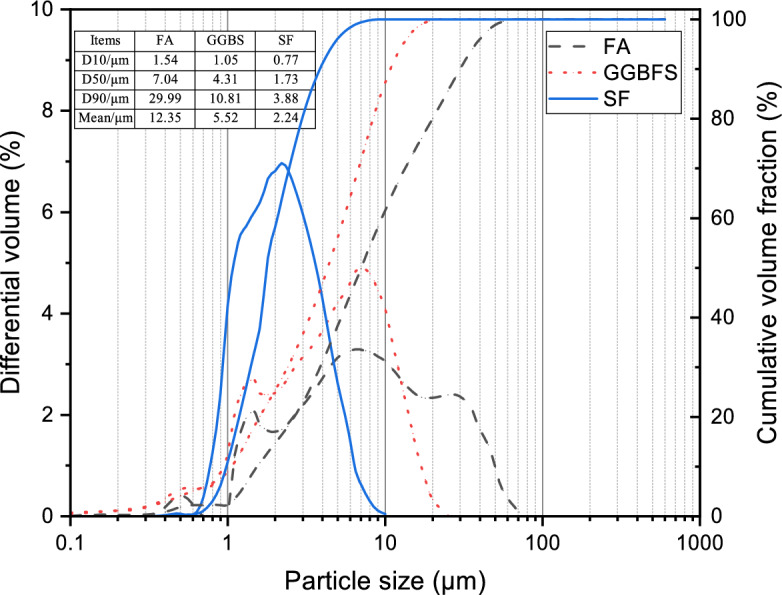
Size distribution curves for GGBFS, FA, and SF particles.

As shown in Fig. 3, laser particle size analysis revealed mean particle diameters of 12.35 µm, 5.52 µm, and 2.24 µm for FA, GGBFS, and SF, respectively. This broad particle size range facilitates a well-graded distribution, promoting improved packing density and the development of a denser composite matrix^11^.

The alkaline activator solution was obtained by combining solid caustic soda (≥ 98% NaOH, Xinjiang Zhongtai Chemical Co., Ltd.) with sodium silicate solution (SS) containing 12.9% Na_2_O and 29.2% SiO_2_ (Hebei Mengxing Chemical Co., Ltd.) and water. The activator solution was produced by dissolving caustic soda flakes in water, mixing with raw SS, then sealing and conditioning at the target temperature for at least 24 h.

**Fig. 4:**
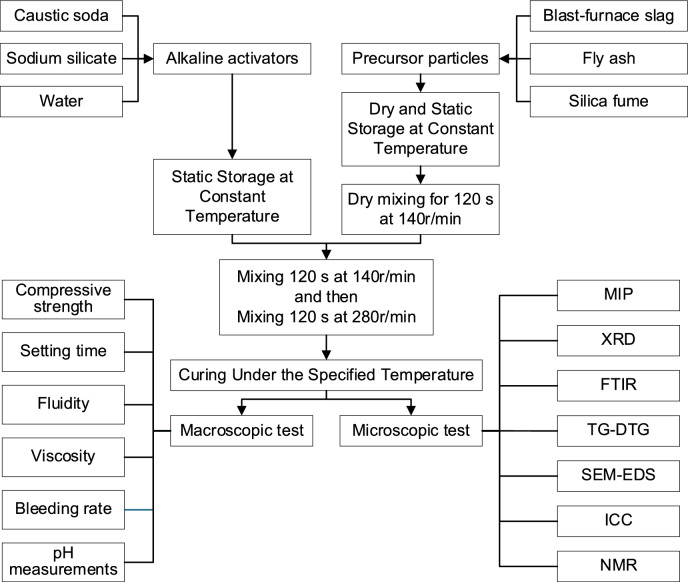
Preparation scheme for the AAGM.

For specimen preparation, precursors were dried and stored at the required test temperature for at least 24 h. Following GB/T 1346-2024, precursors were dry-mixed for 120 s, then the alkaline activator was introduced with stirring at slow speed (120 s) then fast speed (120 s). The resulting AAGM paste was either tested immediately at the required temperature or cured under controlled temperature and humidity conditions (≥ 90% RH) at 20 °C, 40 °C, or 60 °C, as shown in Fig. 4.

### Experimental design

To explore the effects of crucial mix design factors on AAGM performance across a range of curing temperatures, a single-factor experimental design was employed. This approach allows for a systematic assessment of the effect of each individual parameter on material behavior. The data obtained from this study are intended to facilitate the determination of suitable parameter ranges, which will serve as the foundation for future multivariate optimization studies, such as those employing response surface methodology (RSM), to achieve precise performance tailoring.

Five key factors were considered:

Factor *A* (GGBFS ratio in precursor): $A = m_{{{\mathrm{GGBFS}}}} /\left( {m_{{{\mathrm{GGBFS}}}} + m_{{{\mathrm{FA}}}} + m_{{{\mathrm{SF}}}} } \right)$.

Factor *B* (FA ratio in supplementary materials): $B = m_{{{\mathrm{FA}}}} /\left( {m_{{{\mathrm{FA}}}} + m_{{{\mathrm{SF}}}} } \right)$.

Factor *C* (liquid-to-solid ratio): $C = l/s = m_{{\mathrm{l}}} /\left( {m_{{{\mathrm{GGBFS}}}} + m_{{{\mathrm{FA}}}} + m_{{{\mathrm{SF}}}} } \right)$.

Factor *D* (activator modulus): $D = M_{{\mathrm{s}}} = n_{{{\mathrm{(SiO}}_{{2}} {)}}} /n_{{{\mathrm{(Na}}_{{2}} {\mathrm{O}}{)}}}$.

Factor *E* (Na_2_O content in activator): $E = {\mathrm{Na}}_{{2}} {\mathrm{O}}\% = m_{{{\mathrm{(Na}}_{{2}} {\mathrm{O}}{)}}} /m_{{\mathrm{l}}}$.

Where $m_{{{\mathrm{GGBFS}}}}$, $m_{{{\mathrm{FA}}}}$, $m_{{{\mathrm{SF}}}}$, $m_{{\mathrm{l}}}$ and $m_{{{\mathrm{(Na}}_{{2}} {\mathrm{O}}{)}}}$ refer to the masses of GGBFS, FA, SF, the alkaline activator solution, and Na_2_O in the activator solution, respectively; $n_{{{\mathrm{(SiO}}_{{2}} {)}}}$ and $n_{{{\mathrm{(Na}}_{{2}} {\mathrm{O}}{)}}}$ are the molar quantities of SiO_2_ and Na_2_O in the activator.

Each parameter was varied across a wide range to comprehensively evaluate the AAGM performance characteristics. The selection of these ranges was based on a combination of literature review and preliminary experimental feasibility. While numerous prior studies have investigated mix parameters for alkali-activated materials (AAM)^20,21^, those works primarily focus on structural applications. In contrast, AAGM for grouting must meet not only mechanical performance requirements, but also strict demands in terms of fresh-state behavior, such as fluidity, viscosity, and setting time. Therefore, wider factor ranges were employed to fully evaluate performance trade-offs under varying conditions.

To simulate realistic field environments**,** including ambient and deep tunnel/mine scenarios, each experimental group was cured at 20 °C, 40 °C, or 60 °C. In total, 63 distinct mix designs were developed and tested under these conditions. Specimen 20-CE (where “20” corresponds to the curing temperatures of 20 °C) was designated as the central control mix because it represents the intermediate levels of all compositional factors (GGBFS ratio = 0.5, FA/SF ratio = 1, l/s = 0.6, M_s_ = 1.8, Na_2_O% = 9) cured at standard ambient temperature (20 °C), providing a baseline for comparative analysis. Tables 2 and 3 present the detailed compositions and matrix design.

**Table 2:** Factors and levels in experimental design.

Factor	Temperature	GGBFS ratio (*A*)	*m*_FA_/(*m*_FA_ + *m*_GF_) (*B*)	*l/s* (*C*)	*M*_s_ (*D*)	Na_2_O% (*E*)
Level 1	20 °C	0	0	0.3	0	5
Level 2	40 °C	0.25	0.25	0.6	0.6	7
Level 3	60 °C	0.5	0.5	0.9	1.2	9
Level 4	–	0.75	0.75	1.2	1.8	11
Level 5	–	1	1	1.5	2.3	13

**Table 3:** Design of experiment.

No	Temperature (°C)	GGBFS ratio (*A*)	*m*_FA_/(*m*_FA_ + *m*_GF_) (*B*)	*l/s* (*C*)	*M*_s_ (*D*)	Na_2_O% (*E*)
20-A1	20	0	0.5	0.6	1.8	9
20-A2	20	0.25	0.5	0.6	1.8	9
20-CE	20	0.5	0.5	0.6	1.8	9
20-A4	20	0.75	0.5	0.6	1.8	9
20-A5	20	1	0.5	0.6	1.8	9
20-B1	20	0.5	0	0.6	1.8	9
20-B2	20	0.5	0.25	0.6	1.8	9
20-B4	20	0.5	0.75	0.6	1.8	9
20-B5	20	0.5	1	0.6	1.8	9
20-C1	20	0.5	0.5	0.3	1.8	9
20-C3	20	0.5	0.5	0.9	1.8	9
20-C4	20	0.5	0.5	1.2	1.8	9
20-C5	20	0.5	0.5	1.5	1.8	9
20-D1	20	0.5	0.5	0.6	0	9
20-D2	20	0.5	0.5	0.6	0.6	9
20-D3	20	0.5	0.5	0.6	1.2	9
20-D5	20	0.5	0.5	0.6	2.3	9
20-E1	20	0.5	0.5	0.6	1.8	5
20-E2	20	0.5	0.5	0.6	1.8	7
20-E4	20	0.5	0.5	0.6	1.8	11
20-E5	20	0.5	0.5	0.6	1.8	13
40-A1	40	0	0.5	0.6	1.8	9
40-A2	40	0.25	0.5	0.6	1.8	9
40-CE	40	0.5	0.5	0.6	1.8	9
40-A4	40	0.75	0.5	0.6	1.8	9
40-A5	40	1	0.5	0.6	1.8	9
40-B1	40	0.5	0	0.6	1.8	9
40-B2	40	0.5	0.25	0.6	1.8	9
40-B4	40	0.5	0.75	0.6	1.8	9
40-B5	40	0.5	1	0.6	1.8	9
40-C1	40	0.5	0.5	0.3	1.8	9
40-C3	40	0.5	0.5	0.9	1.8	9
40-C4	40	0.5	0.5	1.2	1.8	9
40-C5	40	0.5	0.5	1.5	1.8	9
40-D1	40	0.5	0.5	0.6	0	9
40-D2	40	0.5	0.5	0.6	0.6	9
40-D3	40	0.5	0.5	0.6	1.2	9
40-D5	40	0.5	0.5	0.6	2.3	9
40-E1	40	0.5	0.5	0.6	1.8	5
40-E2	40	0.5	0.5	0.6	1.8	7
40-E4	40	0.5	0.5	0.6	1.8	11
40-E5	40	0.5	0.5	0.6	1.8	13
60-A1	60	0	0.5	0.6	1.8	9
60-A2	60	0.25	0.5	0.6	1.8	9
60-CE	60	0.5	0.5	0.6	1.8	9
60-A4	60	0.75	0.5	0.6	1.8	9
60-A5	60	1	0.5	0.6	1.8	9
60-B1	60	0.5	0	0.6	1.8	9
60-B2	60	0.5	0.25	0.6	1.8	9
60-B4	60	0.5	0.75	0.6	1.8	9
60-B5	60	0.5	1	0.6	1.8	9
60-C1	60	0.5	0.5	0.3	1.8	9
60-C3	60	0.5	0.5	0.9	1.8	9
60-C4	60	0.5	0.5	1.2	1.8	9
60-C5	60	0.5	0.5	1.5	1.8	9
60-D1	60	0.5	0.5	0.6	0	9
60-D2	60	0.5	0.5	0.6	0.6	9
60-D3	60	0.5	0.5	0.6	1.2	9
60-D5	60	0.5	0.5	0.6	2.3	9
60-E1	60	0.5	0.5	0.6	1.8	5
60-E2	60	0.5	0.5	0.6	1.8	7
60-E4	60	0.5	0.5	0.6	1.8	11
60-E5	60	0.5	0.5	0.6	1.8	13

### Test methods

#### Macroscopic property tests

The macroscopic properties of AAGM were assessed using standard procedures. Compressive strength measurements were carried out on prismatic specimens measuring 40 × 40 × 160 mm following curing periods of 3, 7, and 28 days at designated temperatures, conforming to Chinese standards GB/T 50448-2015 and GB/T 17671-2021. The initial setting time (IST) and final setting time (FST) were determined using a Vicat apparatus, following the procedures described in GB/T 1346-2024. Fluidity of the fresh AAGM paste was measured using truncated conical molds (36 × 60 × 60 mm) placed on a glass plate, in accordance with GB/T 8077-2023. Apparent viscosity was measured using an SNB-2 digital rotational viscometer equipped with automatic temperature compensation, with readings maintained within 30–70% of the instrument’s measuring range. The bleeding rate was determined by placing a 100 mL sample of fresh paste in a sealed graduated cylinder and leaving it undisturbed for 2 h; the ratio of separated water volume to the overall sample volume was calculated following GB/T 50448-2015 and GB/T 50080-2016.

#### Microstructural characterization

For microscopic characterization, core samples were extracted from the center of crushed compressive strength specimens to ensure representation. Reaction was halted by immersing the crushed samples (approximately 3–5 mm fragments) in isopropyl alcohol for 24 h, followed by vacuum drying at 40 °C for another 24 h^22^. The dried samples were then ground into fine powder using an agate mortar and passed through a 200-mesh sieve (75 µm) for powder-based analyses (XRD, FTIR, TG–DTG, NMR).

Phase identification was conducted using a Bruker D8 Advance X-ray diffractometer with Cu-Kα radiation (40 kV, 40 mA), scanning from 5° to 70° (2θ) at a step size of 0.02°. Functional groups were identified using a Bruker Tensor 27 FTIR spectrometer, recording spectra in the 400–4400 cm⁻^1^ range with a resolution of 4 cm⁻^1^. Thermal analysis (TG–DTG) was performed using a WRT-11 analyzer, heating samples from 40 to 900 °C at 10 °C/min under nitrogen. Solid-state ^29^Si and ^27^Al MAS NMR spectroscopy was carried out using a Bruker Ascend Aeon 400WB spectrometer. For SEM–EDS analysis, small fractured pieces were gold-coated and examined using an Apreo S HiVac scanning electron microscope at 15 kV to observe morphology and elemental composition. Pore structure was analyzed using an AutoPore IV automatic mercury porosimeter on block samples (approx. 1 cm^3^) cured for 28 days.

#### Reaction kinetics and activator characterization

To investigate reaction behavior and activator reactivity, pH measurement and isothermal conduction calorimetry (ICC) were conducted. The pH values of the alkali activator solutions were measured using a digital pH meter (PHS-3C), with calibration and measurement performed at the target testing temperatures (20 °C, 40 °C, and 60 °C) to ensure accuracy. Heat evolution during reaction was recorded using a TAM Air isothermal heat flow calorimeter. Fresh pastes were pre-conditioned to the target temperatures (20 °C and 60 °C) and sealed in ampoules before testing to continuously monitor heat flow over 72 h. Although strict temperature control was maintained, a brief period of thermal exchange occurred when placing samples into the calorimeter channels. To minimize this error, data from the initial instable period (typically the first 15–30 min) were excluded during data processing to ensure the accuracy of the reaction kinetics analysis.

## Results and analysis

This investigation examined the effects of various factors upon AAGM performance under different temperature conditions. Specimen 20-CE was selected as the control sample for comparative analysis of macroscopic and microscopic test results. The compressive strength, setting time, fluidity, and viscosity of samples with various mix proportions under different temperature curing environments were analyzed systematically, and the experimental data are presented in Table 4.

**Table 4:** Experimental data for compressive strength, setting time, fluidity, and viscosity.

No	Compressive strength (Mpa)			Setting time (min)		Fluidity (cm)	Viscosity (mPa·s)
	3d	7d	28d	IST	FST		
20-A1	0	0	0	509	747	20.6	2299.5
20-A2	25.05	25.9	38.55	29	35	19.45	2750.7
20-CE	40.7	48.45	62.55	26	30	19.4	3046.6
20-A4	43.35	53.8	64.2	23	27	19.1	4770.2
20-A5	57.55	61.4	65.75	19	24	17.65	6303.4
20-B1	33	34.5	45.75	27	32	16	10,543.2
20-B2	33.2	36.8	47.85	26	30	17.5	5853
20-B4	49.25	54.7	75.6	27	31	20.95	2973.1
20-B5	41.8	52.2	63	26	30	22.8	2462.9
20-C1	63.35	80.85	94.95	15	17	–	–
20-C3	26.75	29.9	35.6	32	39	25.65	348
20-C4	18.45	18.5	30.2	38	45	31	115.8
20-C5	11.35	13.25	20.55	41	47	32	76.5
20-D1	13.6	16.95	26.4	72	81	13.55	9975.2
20-D2	31.4	33.7	41.9	212	232	18.1	9279.6
20-D3	39.8	45.8	58.8	22	27	20.9	4214.1
20-D5	0	0	0	696	1171	14.45	4985.2
20-E1	2.5	20.25	31.5	72	76	18.4	2509.7
20-E2	29.2	37.7	55.3	35	40	18.75	2764.1
20-E4	47.55	58.1	70.2	20	27	21.55	3657.9
20-E5	48.7	67.9	75.9	25	35	20.5	4804.1
40-A1	0	0	0	397	710	19.7	2399.6
40-A2	25.35	27.25	44.8	22	28	19.1	2859.4
40-CE	44.05	50.65	63.55	18	24	18.7	3836.3
40-A4	63.2	75.5	80	17	23	18.3	5506.8
40-A5	64.5	85.5	90.15	15	20	17.1	9132.4
40-B1	39.6	44.75	47.2	17	21	15.5	13,192
40-B2	41.95	45.05	56.65	16	23	17.3	6943.9
40-B4	52.35	62.65	78.45	17	24	20.85	3112.5
40-B5	44.5	53.45	64.7	17	23	22.3	2625.1
40-C1	66.35	82	106.5	10	13	–	–
40-C3	28.8	32.95	37.4	26	31	25.45	500.6
40-C4	19	19.55	33.25	28	32	30.5	122.8
40-C5	13.2	16.4	23.15	36	41	30.8	76.7
40-D1	22.1	22.45	29.2	69	76	10.9	13,634
40-D2	33.35	40.65	46.6	187	231	16.85	10,345.2
40-D3	41.6	49.75	61.6	15	23	19.85	4679
40-D5	0	0	0	494	649	13.4	5462.4
40-E1	19.6	24.35	34	58	65	17.7	3121.2
40-E2	32.5	39.6	57.05	23	31	18.1	3347.4
40-E4	48.1	62.4	74.55	16	22	20.4	4644.7
40-E5	54.55	74.85	81.7	18	24	20.2	5860.1
60-A1	0	0	2.2	347	662	19.45	2816.2
60-A2	50.2	52.7	65.85	20	27	18.65	3110.4
60-CE	61.7	64.85	73.8	15	21	18.5	5282.3
60-A4	65.9	87.1	96.95	13	19	18.1	6484.2
60-A5	81.9	95.05	107.4	13	16	16.1	11,344
60-B1	45.2	59.65	67.9	15	19	14.8	24,922.7
60-B2	54.65	64	72.4	16	22	17.1	9269.7
60-B4	71.65	73.05	83.85	16	20	20.6	3362.2
60-B5	71	71.8	81.9	15	19	21.8	2821.6
60-C1	90.1	90.7	111.25	8	11	–	–
60-C3	46.6	47.8	55.7	17	22	25.2	515.6
60-C4	35.8	39.25	40.05	18	25	29.4	150.8
60-C5	28.7	34.6	36.4	22	27	29.6	86.9
60-D1	26.55	29.15	31.35	49	60	10.4	15,266
60-D2	37.5	44.05	46.8	165	214	14.5	11,924.6
60-D3	51	57.25	70.55	13	19	19.65	5568.7
60-D5	0	0	5.2	287	592	12.45	5725.3
60-E1	25.5	32.2	40.35	43	53	17.55	3633.9
60-E2	53.2	55.3	57.2	21	25	17.85	4105.8
60-E4	66.8	69.5	82.1	14	20	20.1	5963.2
60-E5	55.05	82.1	83.95	16	21	20	6160.1

“–” indicates values beyond the measurable range.

### Compressive strength

**Fig. 5:**
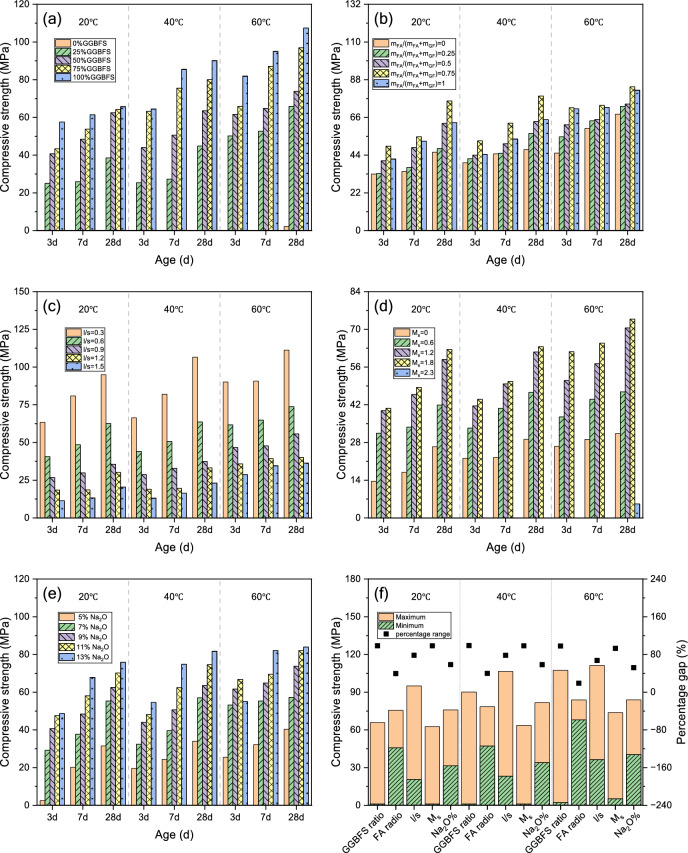
Effects of various factors on compressive strength under different curing temperatures: (**a**) GGBFS ratio; (**b**) m_FA_/(m_FA_ + m_SF_); (**c**) l/s; (**d**) M_s_; (**e**) Na_2_O% and (**f**) The percentage gap between max and min values for every factor.

According to Fig. 5, compressive strength values rose continuously during curing periods owing to progressive reactions and continued formation of reaction products. Consistent with the results of Dai et al.^23^, raising cure temperature from 20 up to 60 °C enhanced compressive strength for all AAGM specimens. This significant strength enhancement is attributed to the accelerated dissolution of precursors and the rapid polymerization of aluminosilicate gels at elevated temperatures, which form a denser microstructure in the early stages. Notably, some samples cured for 3 days at 60 °C exhibited higher strength than identical mix proportions cured for 28 days at 20 °C. This indicates that utilizing these grouting materials at elevated environmental temperatures may achieve superior reinforcement effects and extended service life due to enhanced reaction kinetics.

Under higher temperature curing conditions, the strength differential between samples at varying ages diminished significantly. At 60 °C, the 3-day compressive strength reached approximately 75% of the 28-day strength, demonstrating excellent early strength development. This characteristic is particularly advantageous for emergency reinforcement projects (e.g., mine roof collapse accidents, earthquake disasters, landslide rescue operations) where rapid strength development is critical.

At all temperature conditions, strength increased proportionally with the GGBFS content. Samples without GGBFS exhibited negligible strength except at extended curing periods (28 days) under elevated temperatures (60 °C). This results from GGBFS’s higher CaO content and superior reactivity compared to FA and SF, generating more C–A–S–H phases and producing denser microstructures^24^.

Upon increasing FA content (decreasing SF substitution), strength initially increased before subsequently decreasing. The inflection point occurring at approximately m_FA_/(m_FA_ + m_SF_) = 0.75 indicates that while FA serves as the primary aluminosilicate source, the appropriate inclusion of a small amount of SF (replacing part of FA) enhances AAGM mechanical strength. Excessive SF replacement may lead to agglomeration and increased water demand, while the optimal ratio balances the pozzolanic activity of SF with the workability benefits of FA. The optimal SF replacement ratio ranging between 0 and 0.5, consistent with previous studies^25^.

AAGM strength showed a clear negative correlation with l/s. Under curing conditions of 20 °C, 40 °C, and 60 °C, high-l/s samples (mix proportion C5) demonstrated only 21.6%, 21.7%, and 32.7% of the strength of low-l/s samples (mix proportion C1), respectively. And higher curing temperatures partially mitigated the strength reduction caused by elevated l/s ratios.

At equivalent alkali content, increasing M_s_ from 0 to 0.6, 1.2, and 1.8 resulted in rapid initial strength increases of 59%, 123%, and 137%, respectively. However, the samples with M_s_ = 2.3 (Series D5), the compressive strength was negligible (≈ 0 MPa) and could not be reliably measured under the current testing conditions. This phenomenon is attributed to the reduced pH at high modulus (Table 5), where silicate species exist primarily as highly polymerized oligomers. These stable structures are difficult to depolymerize, thereby hindering the activation of precursors and preventing the formation of a hardened matrix. Consequently, the optimal M_s_ value appears to be approximately 1.8.

**Table 5:** The pH values of sodium silicate with different M_s_ and Na₂O% at various temperatures.

M_s_	Na_2_O%	Temperature-1 (°C)	pH-1	Temperature-2 (°C)	pH-2	Temperature-3 (°C)	pH-3
0	9	20	14.99	40	14.67	60	12.52
0.6	9	20	14.83	40	14.45	60	12.36
1.2	9	20	14.59	40	14.14	60	12.1
1.8	9	20	13.81	40	13.45	60	11.61
2.3	9	20	12.9	40	12.6	60	11.06
1.8	5	20	13.56	40	13.35	60	11.46
1.8	7	20	13.85	40	13.5	60	11.56
1.8	9	20	13.92	40	13.56	60	11.61
1.8	11	20	14.06	40	13.71	60	11.73
1.8	13	20	14.14	40	13.79	60	11.79

Unlike M_s_, alkali equivalent (Na_2_O%) demonstrated a simple positive correlation with compressive strength. As Na_2_O% increased, strength improved continuously, though at a gradually decreasing rate. Notably, for samples with low alkali equivalent (sample E1), increasing curing temperature from 20 to 40 °C and 60 °C significantly enhanced 3-day strength values from 2.5 up to 19.6 as well as 25.5 MPa, representing increases of 784% and 1020%, respectively. Higher curing temperatures substantially improved the early-stage strength development of low-alkali-equivalent AAGM.

The relative influence of various factors on AAGM strength followed the sequence: l/s > M_s_ > m_GGBFS_/(m_GGBFS_ + m_FA_ + m_SF_) > Na_2_O% > m_FA_/(m_FA_ + m_SF_). Elevated curing temperatures effectively mitigated adverse strength effects caused by excessive l/s, insufficient GGBFS content, elevated M_s_, and low alkali equivalent.

### Setting time

**Fig. 6:**
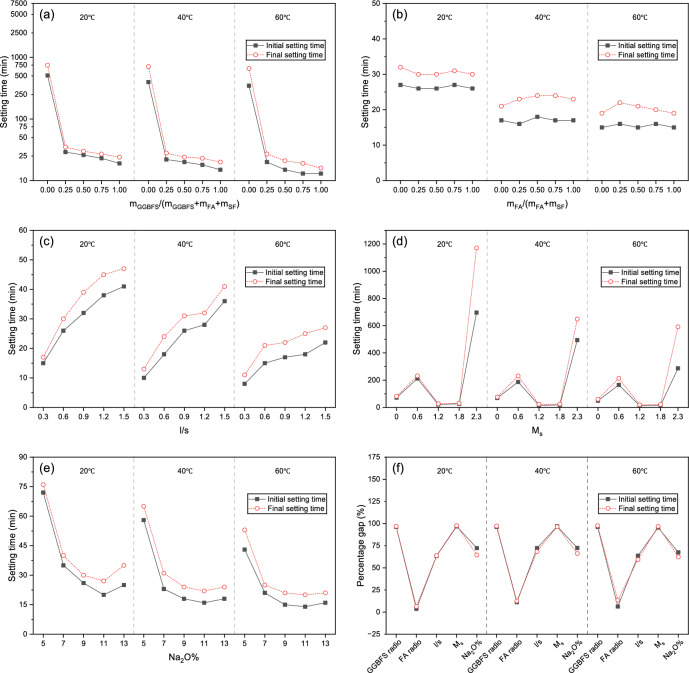
Effects of various factors on setting time under different curing temperatures: (**a**) GGBFS ratio; (**b**) m_FA_/(m_FA_ + m_SF_); (**c**) l/s; (**d**) M_s_; (**e**) Na_2_O% and (**f**) The percentage gap between the maximum and minimum values of each factor.

As shown in Fig. 6, AAGM sets rapidly and shows only a minimal interval between initial and final setting times. This characteristic became more pronounced at elevated temperatures. Increasing curing temperature from 20 to 60°C reduced setting time by 10–50% depending on mix proportions. This rapid solidification property, which is consistent with previous studies^12^, is particularly advantageous for emergency applications where quick formation of effective water barriers is essential. However, this characteristic presents challenges, as excessively rapid setting increases construction complexity. Delayed operations risk slurry solidification within grouting equipment or pipelines, potentially causing blockages and equipment damage. Therefore, controllable setting time represents a critical design parameter for grouting materials.

The influence of individual parameters on setting time is discussed below:

GGBFS Content: Reducing GGBFS proportion effectively extends setting time. When GGBFS content decreased from 25 to 0%, setting time increased from 27–35 to 662–747 min. However, as previously noted, insufficient GGBFS content significantly reduces strength, making this an impractical solution for most applications. Altering the SF/FA ratio had minimal impact on setting time due to their similar reactivity, which was later confirmed by ICC test.

Liquid-to-solid ratio (l/s): Adjusting l/s provides another method for setting time control. Increasing l/s progressively extended setting time. Under all three curing temperatures, the setting time for maximum l/s specimen (sample C5) was approximately five times that from minimum l/s specimen (sample C1). However, excessive l/s values compromise strength development.

Activator Modulus (M_s_): Setting time variation with M_s_ followed a complex pattern, initially increasing, then decreasing, before increasing again. This phenomenon results from combined changes in pH and SiO_3_^2−^ concentration, which will be analyzed in detail in Section "pH value analysis".

Alkali Content (Na2O%): With fixed M_s_, increasing Na_2_O% progressively reduced AAGM slurry setting time until reaching a minimum at Na_2_O% = 11. Further alkali content increases slightly extended setting time.

Effects from different parameters on AAGM setting behavior followed this sequence: M_s_ > l/s > m_GGBFS_/(m_GGBFS_ + m_FA_ + m_SF_) > Na_2_O% > m_FA_/(m_FA_ + m_SF_).

### Fluidity and viscosity

Temperature’s influence on fluidity and viscosity is complex. Higher temperatures increase the Brownian motion of particles, weakening intermolecular forces and theoretically reducing viscosity. However, elevated temperatures simultaneously accelerate GGBFS, FA, and SF dissolution, releasing silicates and aluminates that rapidly form C–(A)–S–H/N–A–S–H 3D colloidal networks, increasing viscosity as well as reducing fluidity^26^. The latter mechanism predominated in our experiments, resulting in increased apparent viscosity and decreased fluidity with rising temperature.

**Fig. 7:**
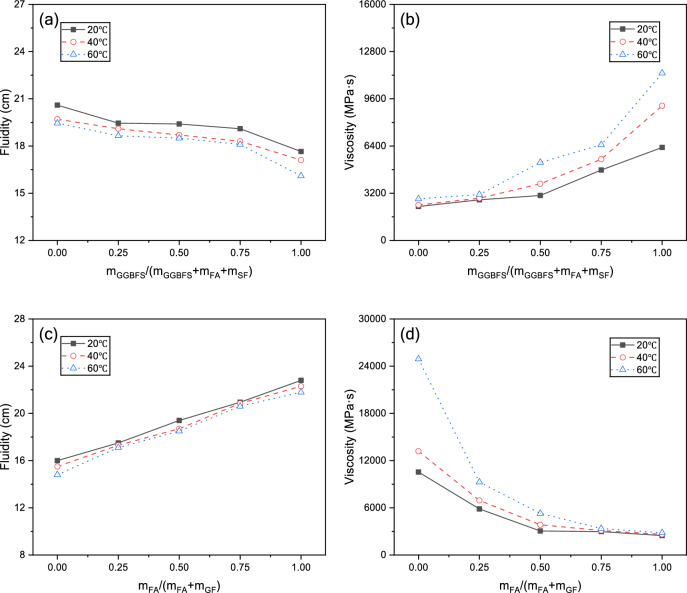

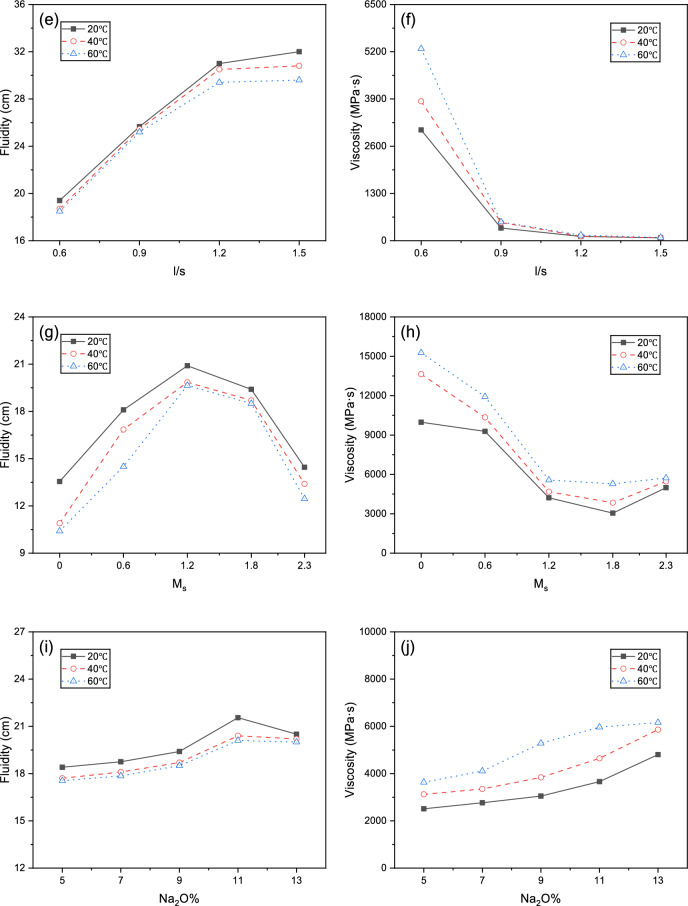
Effects from different parameters upon fluidity and viscosity under different curing temperatures: (**a**, **b**) GGBFS ratio; (**c**, **d**) m_FA_/(m_FA_ + m_SF_); (**e**, **f**) l/s; (**g**, **h**) M_s_; (**i**, **j**) Na_2_O%.

As shown in Fig. 7, under all temperature conditions, increasing GGBFS content increased viscosity and reduced fluidity. Unlike the irregular GGBFS particles, FA and SF feature smooth spherical particles of varying sizes with lower reactivity. This "ball-bearing effect" reduces interparticle friction, decreasing yield stress and plastic viscosity while increasing fluidity^27^. Compared to FA, SF’s extremely fine particles present larger specific surface area, increasing water demand and reducing lubrication efficiency. Consequently, as SF replacement of FA decreases, slurry viscosity decreases and fluidity increases, consistent with previous studies^28^.

Increasing l/s introduces more free water, diluting solid particles and significantly reducing interparticle friction and interaction, substantially decreasing viscosity and increasing fluidity, as reported by Xu et al.^29^. With increasing M_s_, AAGM slurry fluidity initially increased then decreased, with the inflection point near M_s_ = 1.2. Viscosity decreased at first and subsequently increased, exhibiting a transition around M_s_ = 1.8. With increasing Na_2_O%, viscosity continuously increased, while fluidity initially increased before declining, with the turning point at Na_2_O% = 11.

Relative effects from various factors upon AAGM fluidity and viscosity followed the sequence: l/s > M_s_ > m_FA_/(m_FA_ + m_SF_) > m_GGBFS_/(m_GGBFS_ + m_FA_ + m_SF_) > Na_2_O%.

### Bleeding rate

**Fig. 8:**
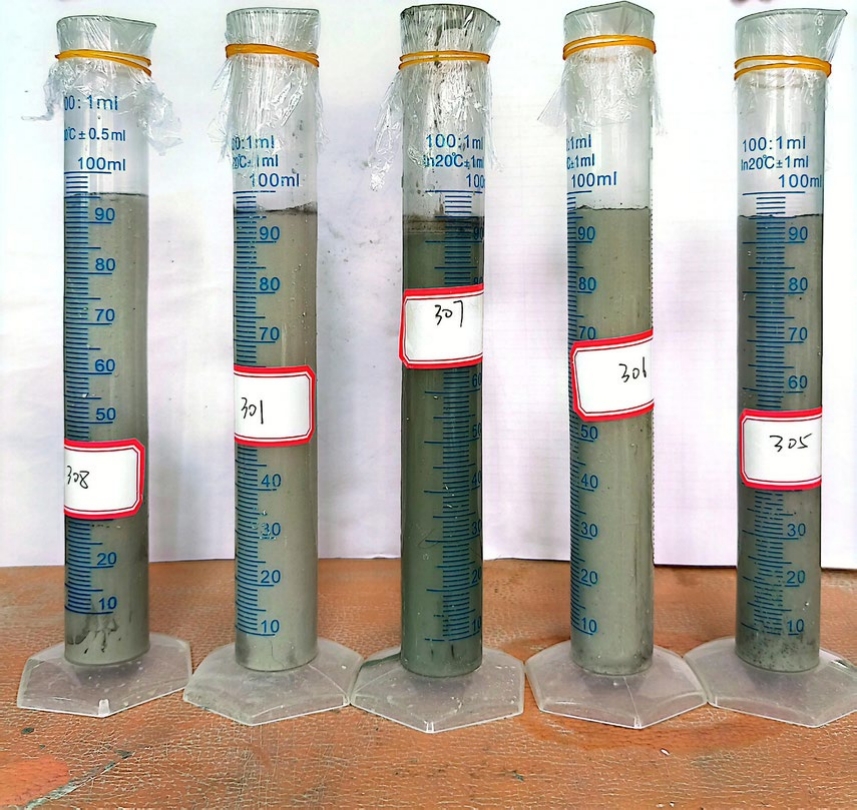
Bleeding rate of AAGM with different proportions and under different curing temperatures.

This characteristic is essential for applications requiring precise filling of voids and fractures. Unlike cement-based grouting materials, which typically require bleeding rates below 5% to ensure acceptable stability, all AAGM samples in this study exhibited bleeding rates below 1%. Specifically, no visible supernatant water layer was observed in the sealed graduated cylinders after the 2-h resting period, indicating excellent stability as illustrated in Fig. 8. This observation agrees with the conclusions drawn by Guo et al.^12^. Consequently, further analysis of bleeding rate variation was deemed unnecessary.

Considering compressive strength, setting time, fluidity, viscosity, and bleeding rate comprehensively, the optimal ranges for AAGM compositional parameters were determined as: m_GGBFS_/(m_GGBFS_ + m_FA_ + m_SF_): 0.5–1, m_FA_/(m_FA_ + m_SF_): 0.5–1, l/s: 0.6–1.2, M_s_: 1.2–2.3, and Na_2_O%: 9–13.

### pH value analysis

**Fig. 9:**
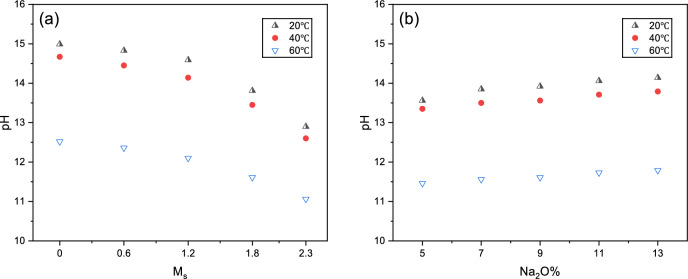
The pH values of sodium silicate with different factors: (**a**) M_s_ and (**b**) Na₂O%.

As Na_2_O% increases, the activator generates more OH⁻ ions, thereby elevating pH and enhancing alkalinity, as shown in Table 5 and Fig. 9. This trend of increasing alkalinity correlates with the accelerated reaction rates observed. This promoted Ca^2+^ and Al^3+^ dissolution from GGBFS and FA, accelerated C–A–S–H phase development, simultaneously enabled high-activity SiO_2_ from SF to participate in reactions, forming dense binder structures that filled pores and progressively enhanced strength. However, excessive Na^+^ concentration caused rapid precipitation of reaction products (“flash setting”), resulting in porous or microcracked structures. Consequently, when Na_2_O% exceeded 11, further alkali content increases significantly slowed strength development, consistent with the findings in Section "Compressive strength".

At constant M_s_, increasing Na_2_O% elevated total ion concentration in the water–glass solution, continuously increasing water–glass viscosity and, consequently, AAGM slurry viscosity. Elevated reaction rates from high alkalinity shortened setting times. However, excessive alkalinity potentially formed dense calcium silicate passivation layers (~ 50 nm thickness) on GGBFS particle surfaces, inhibiting further dissolution. Therefore, when Na_2_O% increased from 11 to 13, setting time slightly decreased. Fluidity improvements resulted from increased zeta potential magnitude on particle surfaces in alkaline environments, enhancing interparticle dispersion. However, at Na_2_O% = 13, excessive viscosity negatively impacted fluidity.

At Na_2_O% = 9, increasing M_s_ from 0 to 2.3 progressively decreased pH of SS and weakened alkalinity. At low M_s_, silicate existed primarily as monomers (SiO_3_^2−^, H_2_SiO_4_^2−^), with hydrolysis maintaining high pH.

1 $${\mathrm{SiO}}_{3}^{2 - } + 3{\mathrm{H}}_{2} {\mathrm{O}} \leftrightarrow {\mathrm{H}}_{4} {\mathrm{SiO}}_{4} + 2{\mathrm{OH}}^{ - }$$

As M_s_ increased, higher SiO_2_ content promoted silicate polymerization (forming species like Si_5_O_10_^2−^, Si_6_O_12_^2−^), reducing hydrolysis tendency and OH^−^ release, thereby decreasing pH. Additionally, partial silicate conversion to neutral silicic acid (H_4_SiO_4_) generated H^+^ that partially neutralized OH^−^, further reducing pH.

Under fixed conditions (Na_2_O% = 9%, M_s_ = 1.8), increasing the temperature from 20 to 60 °C decreased the solution pH because the accelerated polymerization of silicate species consumed free OH⁻ through the formation of higher-order silicate units. Representative reactions include the condensation of silanol groups:

2 $$\equiv {\mathrm{Si}}{-}{\mathrm{OH}} + {\mathrm{HO}}{-}{\mathrm{Si}} \equiv \to \equiv {\mathrm{Si}}{-}{\mathrm{O}}{-}{\mathrm{Si}} \equiv + {\mathrm{H}}_{2} {\mathrm{O}}$$

This process reflects the consumption of OH^−^ during silicate polymerization, rather than the release of water itself being the primary factor affecting alkalinity. Simultaneously, elevated temperatures promoted endothermic H_4_SiO_4_ dissociation, increasing H^+^ concentration and further neutralizing OH^−^.

3 $${\mathrm{H}}_{4} {\mathrm{SiO}}_{4} \leftrightarrow {\mathrm{H}}^{ + } + {\mathrm{H}}_{3} {\mathrm{SiO}}_{4}^{ - }$$

4 $${\mathrm{nSiO}}_{3}^{2 - } + {\mathrm{mH}}_{2} {\mathrm{O}} \leftrightarrow {\mathrm{Si}}_{n} {\mathrm{O}}_{3n - m}^{2m - } + {\mathrm{mOH}}^{ - }$$

Despite reduced activator alkalinity at higher temperatures, accelerated water–glass hydrolysis and silicate ion polymerization enhanced AAGM reaction rates, resulting in shortened setting times, increased strength (particularly early-stage), elevated viscosity, and reduced fluidity. Overall, M_s_ demonstrated more significant impact on pH and slurry reactivity than Na_2_O% under all temperature conditions.

### MIP analysis

**Fig. 10:**
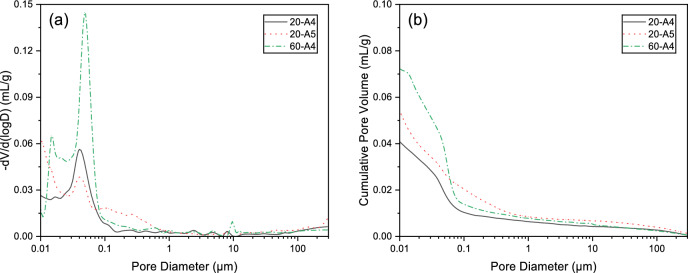
Pore structural properties for AAGM after 28-day curing: (**a**) pore size distribution; (**b**) cumulative pore volume.

The pore structure significantly influences the hardened properties of grouting materials. To ensure a consistent basis for evaluating pore structure evolution, only specimens with controlled variables were selected for detailed comparison. Therefore, the MIP results of samples 20-A4, 20-A5, and 60-A4 were analyzed to examine the effects of slag content and curing temperature, as presented in Table 6 and Fig. 10. Pore size distribution was classified into: Gel pores (G) (0–0.01 µm), Transition pores (T) (0.01–0.05 µm), Capillary pores (C) (0.05–0.1 µm), Macropores (M) (0.1–1 µm), and Air pores (A) (> 10 µm)^30,31^.

**Table 6:** Pore structural properties for AAGM after 28-day curing.

Specimens	Pore size distribution (%)					Total porosity (%)
	< 0.01 μm	0.01–0.05 µm	0.05–0.1 µm	0.1–1 µm	> 1 µm	
20-A4	18.03	44.77	20.29	8.67	8.25	4.85
20-A5	22.36	41.43	10.16	18.98	7.07	7.01
60-A4	22.19	37.47	27.08	7.14	6.12	9.33

The pores in AAGM were predominantly distributed within the 0.002–0.1 µm range, primarily as Transition pores^28,32^. Macropores and Air pores exceeding 0.1 µm resulted from cracks and defects^33^. Comparing samples A4 and A5, the incorporation of FA and SF reduced the reaction rate and decreased crack formation. The fine SF particles filled cracks and provided nucleation sites, significantly reducing the total porosity in sample A4, consistent with the SEM images in Section "SEM–EDS analysis". This transformation of Macropores to Capillary pores contributed to strength enhancement; however, strength test revealed sample A5 outperformed sample A4. This indicates that for AAGM, the positive effect of increased reaction products on strength outweighs the influence of pore structure.

For Sample A4, while the pore size distribution remained similar under both curing temperatures, the total porosity increased from 4.85 to 9.33% at 60 °C, a 92% increase. Nevertheless, the positive effect of increased reaction products at elevated temperature outweighed the negative impact of increased porosity, resulting in higher overall strength. This is because the accelerated polymerization at 60 °C produces a greater volume of high-strength geopolymer gel that effectively bridges the solid particles and reinforces the matrix.

### XRD analysis

**Fig. 11:**
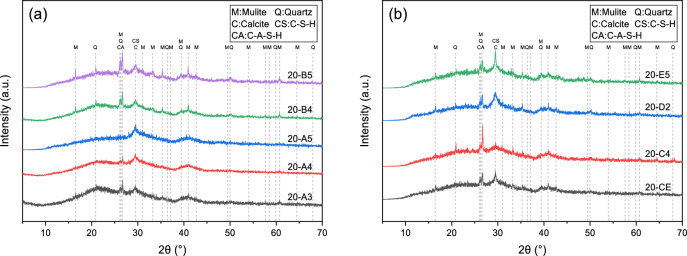
XRD spectra of AAGM specimens cured at 20 °C for 28 days: (**a**) Specimens CE, A4, A5, B4 and B5; and (**b**) Specimens CE, C4, D2, E5.

XRD patterns of different specimens maintained at 20 °C over 28 days are presented within Fig. 11. A common feature observed in all hardened specimens is the occurrence of broad humps between 2θ span from 20° to 40°, demonstrating formation of amorphous aluminosilicate phases after alkali-activation. This amorphous hump corresponds to concurrent presence of C–A–S–H with N–A–S–H silicate phases^34^. In alkali-activated systems, the formation of these amorphous gels is primarily a result of polymerization or condensation reactions rather than traditional hydration as observed in Portland cement. This hump’s shift to a higher 2θ angle compared to raw materials demonstrates dissolution of vitreous phases and subsequent formation of amorphous binding phases^35^. Crystalline diffraction signals from quartz along with mullite originate from FA, and their significant intensity reduction indicates geopolymerization under the influence of the activator.

**Fig. 12:**
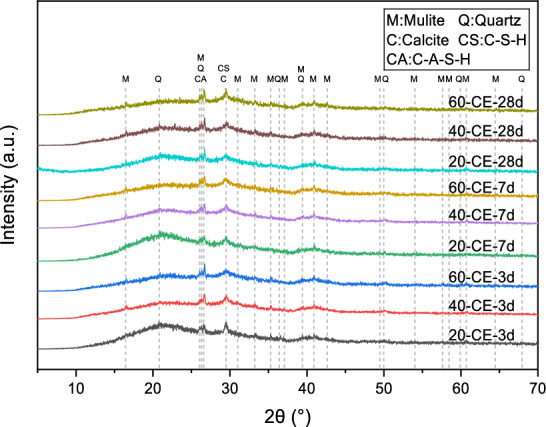
XRD spectra of sample CE at different ages under different curing temperatures.

Figure 12 displays X-ray diffraction spectra of specimen CE across various timepoints under different maintenance temperatures (20 °C, 40 °C, and 60 °C). At 20 °C, from 3 to 28 days, hump intensity within 20°–25° range continuously decreased, while broad peak intensity throughout entire 20°–40° angular range increased. Meanwhile, peaks attributed to mullite and quartz showed reduced intensity, with new humps forming at 26.5° and 29.5°, indicating dissolution of mineral phases and vitreous bodies and formation of C–(A)–S–H and N–A–S–H polymerization products.

With increased curing temperature, the 3-day XRD patterns showed decreasing hump intensity in the 20°–25° range, reduced quartz and mullite crystal diffraction peaks, and increased intensity of the broad hump across the 20°–40° range. The new humps near 26.5° and 29.5° also intensified. Peak variations resulting from elevated curing temperatures resemble effects of prolonged aging. Under enhanced temperature conditions, differences among 3-day and 28-day XRD patterns diminished, indicating that elevated temperatures promoted dissolution of vitreous and mineral phases and accelerated formation of gel products.

Comparing 28-day XRD patterns of mix ratios CE, A4, and A5, increased GGBFS content corresponded with decreased hump intensity in the 20°–25° range and increased hump intensity near 39.5°. This confirms that GGBFS exhibits higher reactivity than FA and SF, facilitating vitreous body dissolution and reaction product formation. In mix ratios CE, B4, and B5, decreased SF proportion resulted in more prominent mullite and quartz diffraction peaks, indicating that appropriate SF substitution enhances reactions.

The 28-day XRD pattern of mix ratio C4 showed slightly lower broad hump intensity and more pronounced mineral-phase diffraction peaks compared to mix ratio CE, attributed to higher l/s ratios increasing distance between precursor particles and diluting the alkali-activation environment. Mix ratio E5 exhibited significantly higher hump intensity near 29.5° than mix ratio CE, demonstrating that higher alkali content in the activator promotes reaction product formation. Mix ratios D2 and CE displayed relatively similar XRD patterns, suggesting that M_s_ changes have multifaceted impacts: decreased modulus increases slurry pH, enhancing precursor dissolution, but reduces active SiO_3_^2−^ content, adversely affecting the reaction.

Calcite peaks were most prominent in sample E5 and sample CE cured at 60 °C for 28 days, originating from raw materials and C–A–S–H decalcification because of atmospheric CO_2_. This indicates that elevated temperature and alkali equivalent accelerate reactions, increase shrinkage cracks, and provide pathways for CO_2_ ingress, as supported by MIP, FTIR, TG–DTG, and SEM characterization results.

### FTIR analysis

**Fig. 13:**
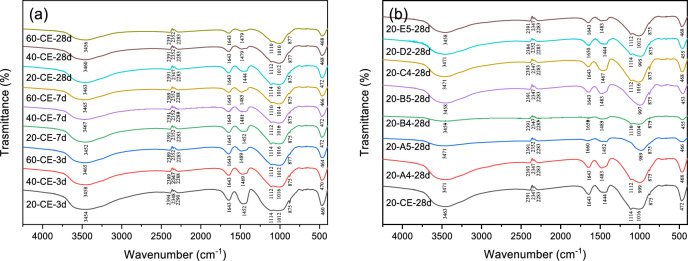
FTIR spectra of AAGM specimens: (**a**) specimen CE across various ages under varying curing temperatures; (**b**) AAGM specimens of various proportions cured at 20 °C for 28 days.

Infrared spectroscopy results for AAGM samples are shown in Fig. 13. Peak positions were similar across mixture ratios with no new peaks forming, confirming that the investigated factors did not produce different reaction products, consistent with X-ray diffraction and thermogravimetric results.

A broad absorption feature around 3450 cm^−1^ and peaks near 1640 cm^−1^ are attributed to –OH stretching and H–O–H bending modes respectively, relating to bound water in reaction products^36^. The signal at approximately 1450 cm^−1^ represents asymmetric stretching of CO_3_^2−^ ions, indicating the presence of carbonates due to atmospheric carbonation^37,38^, which is consistent with the calcite peak near 2θ = 29° in XRD patterns^11,39^.

A signal around 460 cm^−1^ corresponds to Si–O–Si bonds within amorphous glassy bodies of raw materials, and its intensity is independent of crystallization^40^. Absorption peaks in the 800–1200 cm^−1^ range represent asymmetric stretching modes concerning Si–O–T linkages (where T = Si or Al), widely used for studying alkaline aluminosilicate networks^41^. The Si–O–T peak near 875 cm^−1^ indicates incomplete slag reaction^42^, while peaks near 1000 cm^−1^ and 1100 cm^−1^ correspond to Si–O–Si bonds of Q^3^ and Q^4^ silicate groups^11^, corroborated by XRD, MIP, and NMR results.

Comparing FTIR spectra of sample CE cured at different temperatures and ages, peaks near 460 cm^−1^ plus 875 cm^−1^ markedly decreased as curing temperature and age increased. This confirms that higher curing temperatures enhance vitreous body dissolution and polymerization product formation, resulting in shorter setting times, higher strength, increased viscosity, and reduced fluidity, consistent with ICC results.

As GGBFS content increased from sample CE to sample A4 and A5, peak intensities near 1000 cm^−1^ and 1100 cm^−1^ decreased and shifted to higher frequencies. This occurs because excessive GGBFS promotes C–(A)–S–H formation while suppressing N–A–S–H generation, thereby reducing Si–O–Al three-dimensional network structures^43^. Consistent with the ^29^Si NMR results, this phenomenon suggests that suitable addition concerning FA and SF can enhance polymerization degree regarding reaction products. Additionally, sample A5 exhibited significantly smaller peaks at 3450 and 1640 cm^−1^ compared to samples CE and A4, as high-reactivity GGBFS rapidly generates C–(A)–S–H, consuming free water. Nevertheless, C–(A)–S–H is prone to water loss and shrinkage, generating cracks detrimental to mechanical properties.

Sample B4 showed smaller C-O absorption bands around 1450 cm^−1^ compared with samples CE and B5, indicating that appropriate SF content mitigates the negative effects of CO_2_ carbonation on hardened stone body strength. This likely results from SF’s significantly smaller particle size compared to FA and GGBFS, providing a gap-filling effect that reduces adverse impacts of pores and shrinkage cracks, forming a more compact structure that impedes CO_2_ penetration, as confirmed by SEM imaging.

Sample C4 exhibited generally lower peak intensities across the spectrum compared to sample CE, as increased l/b ratios disperse solid particles, reducing their concentration per unit volume and consequently decreasing chemical bond quantity and sample absorbance. Samples D2 and E5 showed lower peak intensities at 3450 cm^−1^, 1640 cm^−1^, 875 cm^−1^, and 460 cm^−1^ compared with sample CE, as decreased M_s_ and increased Na_2_O% enhance activator alkalinity, facilitating depolymerization of Si–O–Si and Al–O–Al bonds in the precursor, promoting vitreous body dissolution and reaction progress, and consuming more free water (pore solution). However, Sample D2 did not show significantly enhanced peaks near 1000 cm^−1^ and 1100 cm^−1^, as decreased M_s_ reduces active SiO_3_^2−^ concentration in the activator, adversely affecting reaction.

### TG–DTG analysis

**Fig. 14:**
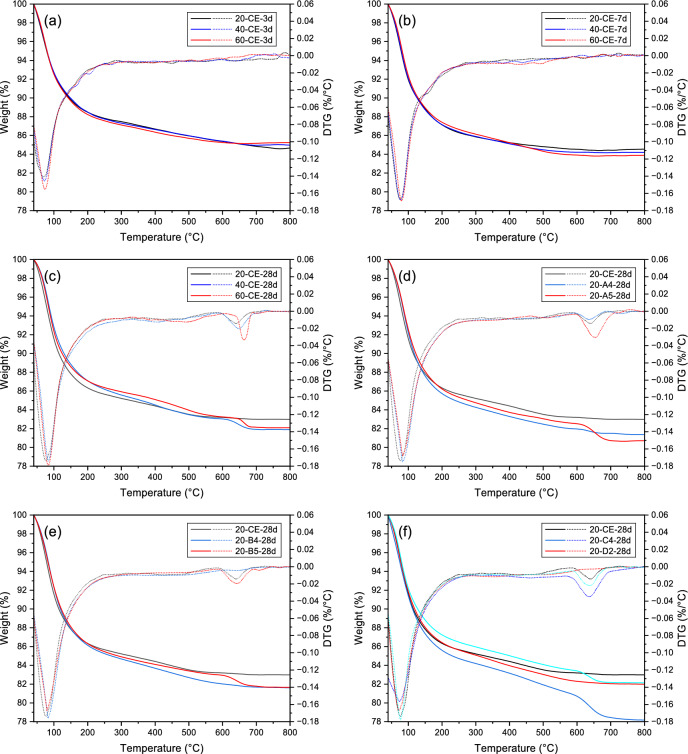
TG–DTG curves of AAGM specimens: (**a**)–(**c**) specimen CE at different ages under different curing temperatures; and (**d**)–(**f**) AAGM specimens with various proportions cured at 20 °C for 28 days.

TG–DTG curves of AAGM specimens are presented in Fig. 14, with figures (a)–(c) showing sample CE curves at different curing temperatures and ages, and figures (b)–(f) displaying curves for different specimens after 28 days of curing during standard conditions.

Two distinct endothermic peaks appear in all specimens.

The first peak occurs at 40–200 °C, primarily resulting from dehydration of C–(A)–S–H and N–A–S–H phases^44^. With increasing curing temperature from 20 to 60 °C, sample CE at the same age showed increased peak values and shifted peak positions to higher temperatures, indicating increased reaction product formation and improved thermal stability^45^. Higher peak temperatures typically signify greater polycondensation degree in gel products, requiring increased energy for water molecule release from the inorganic polymer framework^46^, consistent with ^29^Si NMR results.

The second peak appears at 600–800 °C, corresponding to CaCO_3_ decarbonation. Notably, this peak became increasingly prominent with higher curing temperatures and longer ages, as accelerated C–A–S–H formation induced the development of shrinkage cracks, which acted as pathways for atmospheric CO_2_ ingress, ultimately promoting carbonation. This result is supported by both XRD patterns and SEM observations. However, as mentioned previously, while elevated temperatures may induce microcracking and shrinkage (as noted in the MIP section), the overall benefit of accelerated gel formation dominates the strength development.

Sample CE exhibited a lower first peak intensity than sample A4, indicating that excessive replacement of GGBFS with FA and SF decreases calcium levels and the amount of generated C–A–S–H phase. Sample A5 also showed lower first peak intensity than sample A4, while its second peak intensity was significantly higher. This occurs because FA and SF reaction generates N–A–S–H, which mitigates C–A–S–H shrinkage cracks and reduces negative carbonation effects on reaction products. Similar observations were made when comparing samples CE, B4, and B5, with Sample B4 showing the most prominent first peak and smallest second peak. This is attributed to SF’s significantly smaller particle size compared to FA and GGBFS, with its “micro-aggregate effect” enhancing homogeneity and compactness of reaction products while reducing adverse effects like carbonation.

Sample C4 showed a lower temperature corresponding to the first peak compared to sample CE, as increased l/s ratios augment free water (pore solution) proportion in the inorganic polymer framework, and free water typically evaporates at lower temperatures than bound water. Sample D2, with lower M_s_ than sample CE, contained fewer reactive silicate ions, resulting in slower reaction kinetics and formation of a denser reaction product matrix more resistant to CO_2_ penetration. Consequently, Sample D2 exhibited a less prominent second decarbonation peak (600–800 °C), consistent with its prolonged setting time and reduced heat release in ICC tests. Sample E5 showed a more pronounced first peak than sample CE, as increased alkali equivalent enhances reaction product formation.

### SEM–EDS analysis

**Fig. 15:**
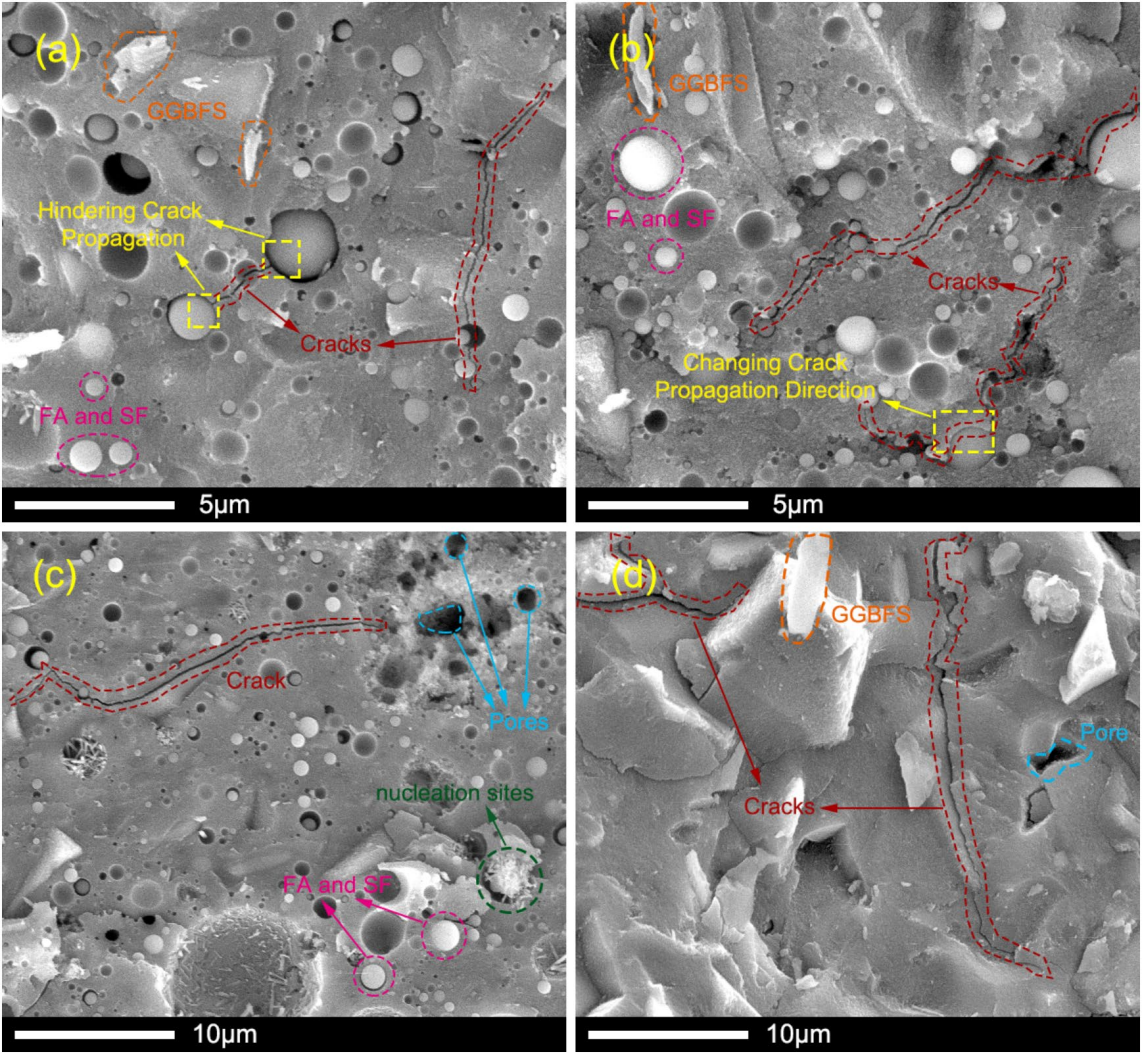
SEM micrographs of AAGM specimens at the age of 28 days: (**a**) 20-CE; (**b**) 40-CE; (**c**) 60-CE and (**d**) 20-A5.

**Fig. 16:**
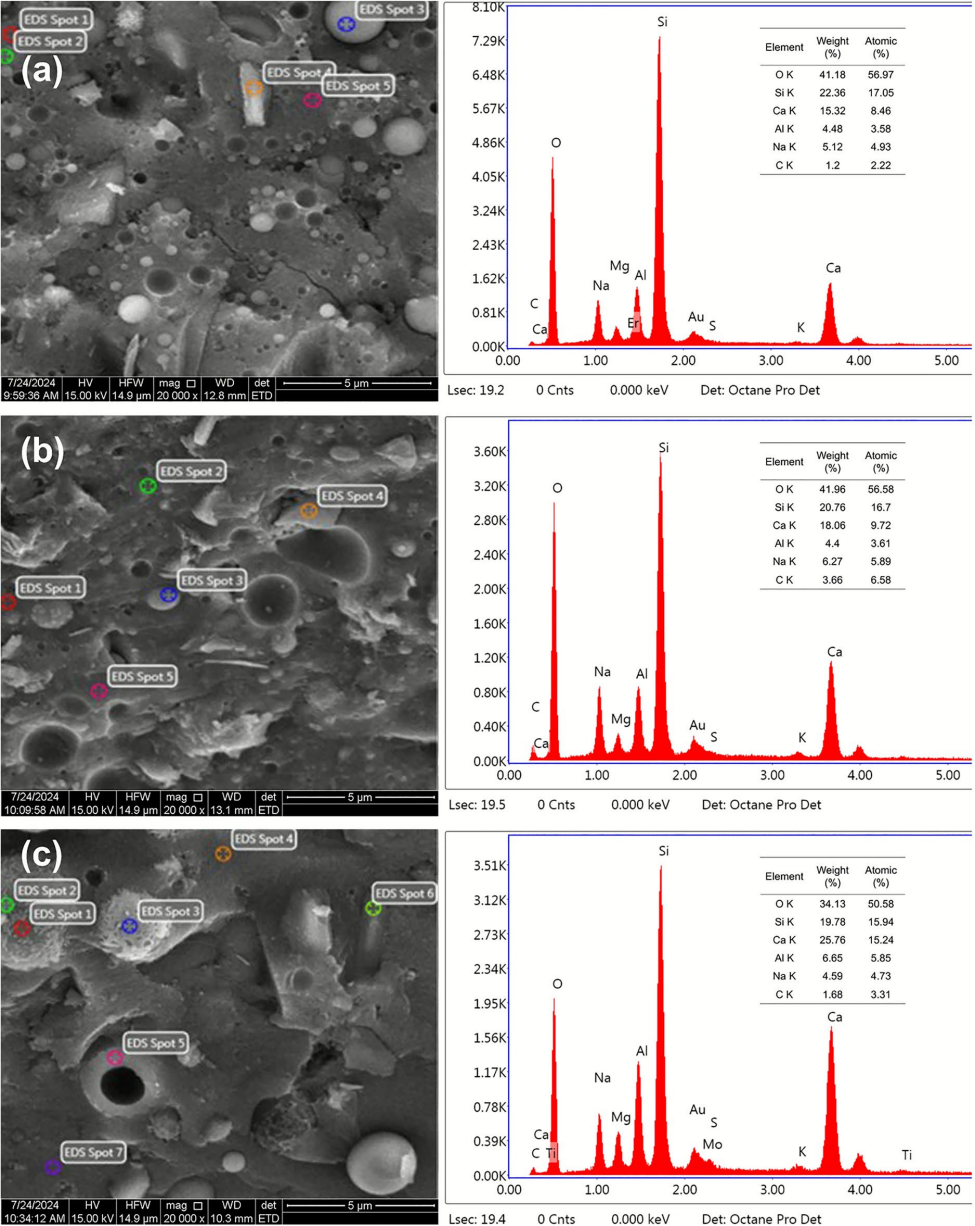
SEM–EDS micrographs of AAGM specimens at the age of 28 days: (**a**) 20-CE; (**b**) 20-B4 and (**c**) 20-B5.

SEM and EDS were employed to characterize microstructural features and elemental composition of AAGM specimens cured for 28 days. Figure 15 illustrates SEM micrographs of Specimens CE and A5 cured at different temperatures, while Fig. 16 presents SEM–EDS images of Specimens CE, B4, and B5 cured at 20 °C for 28 days.

The microstructural analysis revealed that the stone body comprises continuous aluminosilicates interspersed with unreacted or partially reacted FA, SF, and GGBFS particles. Sample CE exhibited microcracks attributable to drying shrinkage, with this phenomenon intensifying at elevated curing temperatures. Elevated temperatures accelerated the reaction rate, but also increased the risk of shrinkage cracking, a phenomenon that should be carefully managed in engineering applications. Under identical curing conditions, Sample CE displayed significantly fewer shrinkage cracks than Sample A5, attributed to the lower CaO content and reduced reactivity of fly ash and silica fume relative to GGBFS. Adding SF and FA into GGBFS effectively reduced the reaction rate and macropore formation, consistent with MIP and ICC test results. Additionally, fine FA and SF particles were observed filling interstitial spaces between cracks, thereby hindering crack propagation and altering crack direction paths, consistent with previous research^25,47,48^.

EDS analysis identified continuous dark gray areas as reaction products, irregular light gray areas as unreacted GGBFS particles, and spherical light gray areas as FA and SF particles^24^. For each AAGM sample, five distinct spots representing the reaction products were randomly selected for EDS acquisition, and the average values were used to determine the elemental composition (Ca, Si, Al, Na) presented in Fig. 16. Elemental analysis verified the existence of O, Na, Al, Si, Ca across all samples, indicating reaction product formation including C–S–H, C–A–S–H, N–A–S–H phases. AAM mechanical properties typically show inverse relationship with Ca/Si ratio while exhibiting direct relationship with Si/Al ratios^49^. With SF replacement of FA decreasing across mixture ratios CE, B4, and B5, Si content in reaction products declined significantly, while Ca and Al contents increased slightly. The Ca/Si ratios for mixture ratios CE, B4, and B5 were 0.50, 0.58, and 0.96, respectively, while the corresponding Si/Al ratios were 4.76, 4.62, and 2.72. Notably, excessive SF content tends to cause particle agglomeration due to its small particle size, which adversely affects strength development. The optimal SF content was determined to be approximately 8%, aligning with strength test results and previous research findings^28^.

### ICC analysis

**Fig. 17:**
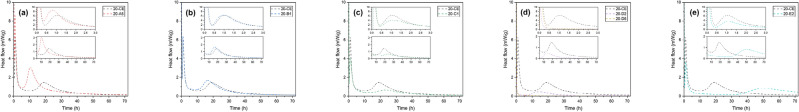
Heat flow curves of specimens cured at 20 °C under different factor conditions: (**a**) GGBFS radio; (**b**) FA ratio; (**c**) l/s; (**d**) M_s_; (**e**) Na_2_O%.

**Fig. 18:**
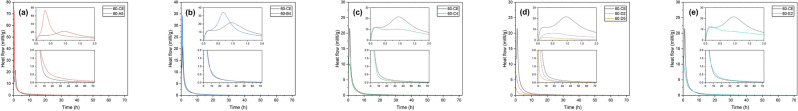
Heat flow curves of specimens cured at 60 °C under different factor conditions: (**a**) GGBFS radio; (**b**) FA ratio; (**c**) l/s; (**d**) M_s_; (**e**) Na_2_O%.

**Fig. 19:**
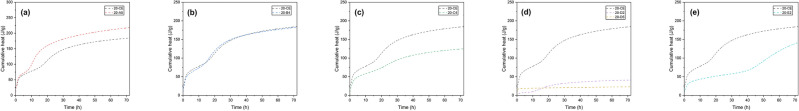
Cumulative heat curves of specimens cured at 20 °C under different factor conditions: (**a**) GGBFS radio; (**b**) FA ratio; (**c**) l/s; (**d**) M_s_; (**e**) Na_2_O%.

**Fig. 20:**
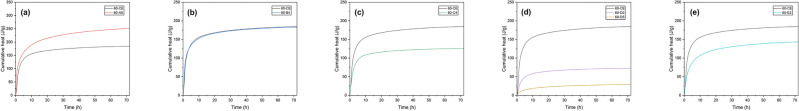
Cumulative heat curves of specimens cured at 60 °C under different factor conditions: (**a**) GGBFS radio; (**b**) FA ratio; (**c**) l/s; (**d**) M_s_; (**e**) Na_2_O%.

Figures 17, 18, 19 and 20 present heat flow curves and cumulative heat curves for AAGM paste specimens at 20 °C and 60 °C, with specimens 20-CE and 60-CE serving as control groups. The thermal evolution of AAGM paste typically progresses through five distinct stages: dissolution, induction, acceleration, deceleration, and steady state^23,50^. The initial peak occurring within 0.25 h represents heat exchange between the instrument and external environment and was excluded from analysis.

At 20 °C, two exothermic peaks were observed. The first peak appeared within the initial 60 min, representing precursor wetting and dissolution. The timing of the second peak varied considerably and correlated with microstructural development. Both peaks occurred earlier and with higher intensity in Specimen A5 compared to the control group, attributable to enhanced reactivity from increased GGBFS content. Specimen B4 exhibited similar behavior to the control group, indicating minimal effect of FA-SF substitution ratio on reaction kinetics.

Specimens C4 and E2 demonstrated delayed peak appearance and significantly reduced peak intensities compared to the control group. This implies that higher liquid/solid ratios increase interparticle spacing, reducing reaction rates and heat evolution. Similarly, insufficient alkali content negatively impacts precursor dissolution and reaction kinetics. Specimen D2 showed considerably delayed and diminished peak intensity, attributed to reduced dissolved silica concentration and decreased reaction rate due to lower M_s_. Specimen D5 displayed no discernible peak, as excessively high M_s_ reduced activator alkalinity, inhibiting precursor dissolution and reaction, consistent with pH test results.

At 60 °C, precursor dissolution and reaction processes were accelerated substantially. The first peak appeared within 0.25 h, the second peak occurred within 2 h, and early-stage cumulative heat release increased significantly, corroborating the setting time test results.

### NMR analysis

**Fig. 21:**
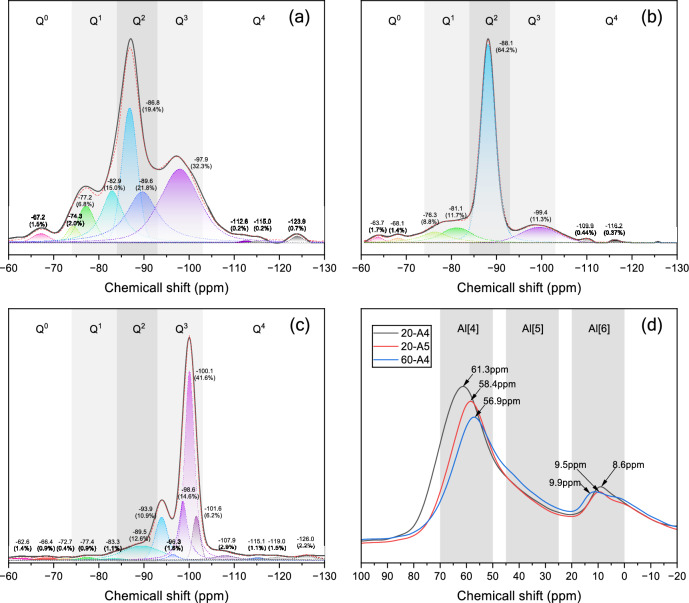
^29^Si and ^27^Al MAS NMR spectra of AAGM specimens: (**a**–**c**) ^29^Si spectra for 20-A4, 20-A5, and 60-A4; (**d**) ^27^Al MAS NMR spectrum.

Peak-fitting of ^29^Si MAS NMR spectra was performed for AAGM specimens, and ^27^Al MAS NMR spectra were compiled for representative specimens, as shown in Fig. 21. Spectral deconvolution was performed using MestReNova software (v15.0.0, Mestrelab Research) by fitting overlapping peaks with mixed Gaussian–Lorentzian line shapes. Peak positions were assigned based on literature-reported chemical shifts for Q^n^(mAl) species^51,52^, and the relative abundance of each structural unit was quantified from integrated peak areas (R^2^ > 0.98). The notation Q^n^(mAl) characterizes the silicon chemical environment, where n (0–4) indicates the quantity of oxygen atoms bridging individual [SiO_4_] tetrahedra with neighboring tetrahedra, with lower n values reflecting diminished [SiO_4_] polymerization. The parameter m (0−n) represents the count of surrounding [AlO_4_] tetrahedra linked to the core [SiO_4_] tetrahedron^53–55^.

Table 7 presents the expected ^29^Si NMR chemical shifts for AAGM specimens based on previous research^51,52^. Statistical analysis of Q^n^ values was conducted, and the mean molecular chain length (MCL) and main chain length of C–(N)–A–S–H phases under cross-linked conditions (MCLC) were calculated using Eq. (5)^56,57^ and Eq. (6)^28,58^, respectively. These parameters were calculated to quantitatively evaluate not only the degree of polymerization but also the connectivity and cross-linking density of the aluminosilicate gel network, providing an integrated structural indicator that directly correlates with mechanical strength development.

5 $$MCL = \frac{{2\left( {Q^{1} + Q^{2} (0{\mathrm{Al}}) + 1.5Q^{2} (1{\mathrm{Al}}) + Q^{3} (0{\mathrm{Al}}) + Q^{3} (1{\mathrm{Al}})} \right)}}{{Q^{1} }}$$

6 $$MCLC = \frac{{\left( {Q^{1} + Q^{2} (0{\mathrm{Al}}) + Q^{2} (1{\mathrm{Al}}) + Q^{3} (0{\mathrm{Al}}) + 2Q^{3} (1{\mathrm{Al}})} \right)}}{{0.25Q^{1} }}$$

**Table 7:** Predicted ^29^Si NMR chemical shifts values for of AAGM (negative shifts, expressed in ppm).

Site type	Chemical shift (ppm)	Site type	Chemical shift (ppm)	Site type	Chemical shift (ppm)
Q^0^	66–73	Q^3^ (0Al)	95–101	Q^4^ (0Al)	103
Q^1^ (0Al)	76–83	Q^3^ (1Al)	~ 95	Q^4^ (1Al)	97–105
Q^1^ (1Al)	~ 75	Q^3^ (2Al)	~ 90	Q^4^ (2Al)	92–99
Q^2^ (0Al)	86–91	Q^3^ (3Al)	~ 85	Q^4^ (3Al)	88–94
Q^2^ (1Al)	~ 85			Q^4^ (4Al)	83–87
Q^2^ (2Al)	~ 80				

Typically, unreacted slag contains numerous Q^0^ sites. Q^1^ sites occur at reaction product chain termini, primarily as short-chain dimers. Q^2^ sites occupy positions within long-chain reaction products, signifying transition from short to long chains. Q^3^ and Q^4^ sites indicate spatial network structure formation in the gel products. Table 8 illustrates that, relative to specimen 20-A5, the optimal incorporation of FA and SF in Specimen 20-A4 provided nucleation sites, increased the degree of polycondensation, and enhanced precursor particle dissolution. Consequently, proportions of Q^0^, Q^1^ and Q^2^ decreased while Q^3^ and Q^4^ increased, forming three-dimensional spatial networks beneficial for strength enhancement. This confirms that the distribution of silicon sites primarily reflects the evolution of the aluminosilicate network structure, rather than merely indicating the presence of reaction products.

**Table 8:** Si (Q^n^) structural proportions with corresponding MCL and MCLC results.

Specimens	Q^0^ (%)	Q^1^ (%)	Q^2^ (%)	Q^3^ (%)	Q^4^ (%)	MCL	MCLC
20-A4	1.47	8.80	34.50	54.09	1.14	23.85	54.20
20-A5	3.13	8.82	75.93	11.30	0.81	23.10	43.55
60-A4	2.28	1.36	13.69	52.54	30.14	100.34	231.35

In Specimen 60-A4, compared to Specimen 20-A4, substantial transformation occurred from low-polymerization forms (Q^1^ and Q^2^) to high-polymerization forms (Q^3^ and Q^4^). Both MCL and MCLC values increased significantly with elevated curing temperature, positively influencing mechanical properties.

The ^27^Al NMR spectra exhibited distinct peaks at 50–70 ppm and 0–20 ppm, with a broad hump at 25–45 ppm, corresponding to 4-coordinated, 6-coordinated, and 5-coordinated aluminum, respectively. Four-coordinated aluminum represents [AlO_4_] tetrahedra substituting for [SiO_4_] in C(N)–A–S–H. Around 60 ppm, four-coordinated aluminum in Sample 20-A4 shifted to lower chemical shifts compared to Sample 20-A5, indicating higher surrounding Si content, decreased electron density, reduced Q^2^(1Al) groups, and increased Q^3^(1Al) groups in C(N)–A–S–H, consistent with ^29^Si NMR analysis. Enhanced peak intensities for four-coordinated and six-coordinated aluminum in Specimen 20-A4 compared to Specimen 20-A5 suggest that SF and FA addition facilitated greater precursor participation in the reaction process.

## Conclusion

This study systematically investigated the temperature-dependent behavior of alkali-activated grouting materials (AAGM) synthesized from industrial waste precursors. By integrating macroscopic testing with multi-scale microstructural characterization, the following key conclusions and engineering insights are drawn:

1. Temperature-Driven Polymerization: Elevated curing temperatures (40–60 °C) significantly accelerate precursor dissolution and the polycondensation kinetics of the aluminosilicate gel. NMR spectroscopy confirms a temperature-induced structural shift from low-coordination species (Q^1^, Q^2^) to high-coordination networks (Q^3^, Q^4^), which is the fundamental mechanism responsible for the rapid development of early-age strength (reaching 75% of 28-day strength in 3 days at 60 °C).
2. Compositional Trade-offs: AAGM performance is governed by the synergistic effects of precursors. While GGBFS governs the setting time and early strength, the incorporation of FA and SF is essential for regulating rheology and refining the pore structure. Specifically, the “ball-bearing effect” of spherical FA particles improves fluidity, while SF acts as a micro-filler. However, excessive activator modulus (M_s_ > 1.8) leads to a highly polymerized silicate environment that inhibits reaction kinetics, resulting in negligible strength development.
3. Optimal Engineering Parameters: To balance the competing requirements of fluidity, setting time, and mechanical strength for underground grouting applications, the optimal compositional ranges are identified as: GGBFS proportion of 0.5–1.0, FA/SF ratio of 0.5–1.0, l/s of 0.6–1.2, M_s_ of 1.2–2.3, and Na_2_O% of 9–13%.
4. Limitations and Recommendations: While high-temperature curing is advantageous for emergency reinforcement due to rapid strength gain, microstructural analysis (MIP and SEM) reveals that it also induces coarser porosity and microcracking due to drying shrinkage. Therefore, in practical engineering applications involving high geothermal temperatures, measures to mitigate shrinkage (such as internal curing agents or fiber reinforcement) should be considered to ensure long-term durability.

## Data Availability

The datasets used and/or analyzed during the current study are available from the corresponding author on reasonable request.
